# An Emerging Approach for Parallel Quantification of Intracellular Protozoan Parasites and Host Cell Characterization Using TissueFAXS Cytometry

**DOI:** 10.1371/journal.pone.0139866

**Published:** 2015-10-21

**Authors:** Maximilian Schmid, Bianca Dufner, Julius Dürk, Konstanze Bedal, Kristina Stricker, Lukas Ali Prokoph, Christoph Koch, Anja K. Wege, Henner Zirpel, Ger van Zandbergen, Rupert Ecker, Bogdan Boghiu, Uwe Ritter

**Affiliations:** 1 Institute of Immunology, University of Regensburg, Regensburg, Germany; 2 Department of Gynecology and Obstetrics, University of Regensburg, Regensburg, Germany; 3 Division of Immunology, Paul-Ehrlich-Institute, Langen, Germany; 4 Institute of Immunology, University Medical Center of the Johannes Gutenberg University of Mainz, Mainz, Germany; 5 TissueGnostics GmbH, Vienna, Austria; Centre National de la Recherche Scientifique, FRANCE

## Abstract

Characterization of host-pathogen interactions is a fundamental approach in microbiological and immunological oriented disciplines. It is commonly accepted that host cells start to change their phenotype after engulfing pathogens. Techniques such as real time PCR or ELISA were used to characterize the genes encoding proteins that are associated either with pathogen elimination or immune escape mechanisms. Most of such studies were performed *in vitro* using primary host cells or cell lines. Consequently, the data generated with such approaches reflect the global RNA expression or protein amount recovered from all cells in culture. This is justified when all host cells harbor an equal amount of pathogens under experimental conditions. However, the uptake of pathogens by phagocytic cells is not synchronized. Consequently, there are host cells incorporating different amounts of pathogens that might result in distinct pathogen-induced protein biosynthesis. Therefore, we established a technique able to detect and quantify the number of pathogens in the corresponding host cells using immunofluorescence-based high throughput analysis. Paired with multicolor staining of molecules of interest it is now possible to analyze the infection profile of host cell populations and the corresponding phenotype of the host cells as a result of parasite load.

## Introduction

Parasite host cell interactions represent one of the most interesting aspects in the field of microbiology and immunology. Since the generation of monoclonal antibodies and the use of fluorochrome labeled reagents, the knowledge about the modification in the biological program of host cells increased steadily.

Indeed, very good immunofluorescence-based approaches to evaluate the parasite host cell interaction exist. Most of the analyses dealing with parasite host cell interactions were performed using multi channel flow cytometry analysis and immunofluorescent microscopy.

Both techniques show experimental advantages and disadvantages. FACS-analyses for example can be used to measure the phenotype of host cells that harbor parasites [1]. However, based on technical limitations it is not possible to analyze the exact number of parasites within the single cells. Furthermore, FACS-analyses are performed with single cell preparations. Consequently, the *in situ* localization of the cells cannot be determined. For that purpose immunefluorescent-based microscopically techniques are more appropriate. Additionally, host cells harboring parasites can be described regarding the expression of certain surface markers *in situ*. However, an objective discrimination of host cells regarding the intensities of markers of interest in context with the parasite load is hardly possible so far. Consequently, a technique useful for quantification of intracellular pathogens and phenotyping of host cells in parallel is needed.

Here we present a method suitable for the detection and quantification of pathogens within the corresponding host cells in parallel using immunofluorescence-based high throughput analysis. With this approach it is now possible to analyze the phenotype of host cells in the context of parasite load.

In our study we have chosen the obligatory intracellular parasite *Leishmania* (L.) *major*. These parasites have evolved a smart strategy to hide from the host immune response once they have entered the dermal compartment: the parasites allow themselves to be absorbed by host cells. Especially phagocytic cells such as macrophages can engulf the pathogen by distinct mechanisms ([2, 3]). Different surface receptors and molecules such as Fc receptors and CD18 are considered to be involved in the internalization of the parasites by macrophages [4, 5]. After phagocytosis, *L*. *major* parasites are detectable within specialized compartments called parasitophorous vacuoles (PV), in which promastigote parasites lose the external flagellum and transform into amastigotes which are adapted to the conditions within the PVs [6]. For the elimination of the parasites, an adaptive T helper (TH) 1 meditated immune response needs to be initiated that is characterized by an early IFN-γ production and the release of leishmanicidal molecules, such as nitric oxide radicals (NO•) by activated macrophages [7]. Otherwise, *L*. *major* parasites are able to hide and amplify inside of these phagocytes.

In the present manuscript we used the TissueQuest and StrataQuest software (TissueGnostics GmbH, Vienna, Austria) including the novel Dot-Finder algorithm for high throughput microscope-based multicolor cell analysis of *L*. *major*-infected macrophages *in situ* and *in vitro*. This procedure allows the detailed analysis of nucleated cells harboring living or dead parasites. Comparable to FACS-based analysis, the expression intensity of fluorescent-labeled markers can be quantified on a single cell level *in situ*. There is no need to create cell suspension, as it is necessary for ImageStream of FACS-analysis. In addition, TissueQuest has also a final validation step in form of a backward gating (from the data back to the cell in the image). This allows the detection of the gated cell subsets *in situ* and gives additional information about the localization of those cells. Furthermore, the backward gating tool can also be applied for final result conformation.

## Materials and Methods

### Ethic statement

All animal work was approved by the local veterinary authorities from “The District Government of Upper Palatinate/Bavaria” based on the international European guidelines and national regulations of the German animal protection act (permission no. 54-2532.1-05/11). Human peripheral blood mononuclear cells (hPBMCs) were isolated from buffy coats (DRK-Blutspendedienst Hessen GmbH, permission no. 329/10). The donors were healthy German adult volunteers without known exposure to *Leishmania* parasites. The named institutional animal care and use committee and ethics committee specifically approved this study.

### Preparation and labeling of *L*. *major* parasites

Virulent *L*. *major* parasites (MHOM/IL/81/FE/BNI) were propagated *in vitro* in blood agar cultures as described previously [8, 9]. Stationary phase promastigotes from the third to seventh *in vitro* passage were harvested, washed four times, and resuspended in PBS (PAN Biotech). Early growth stage logarithmic parasites were used as a model for living parasites and were labeled with 4 μM carboxyfluorescein succinimidyl ester (CFSE). Late growth stage stationary phase parasites were used as a model for apoptotic and dying parasites and were labeled with 4 μM succinimidylester (SE) Alexa Fluor 647 (Life Technologies, Darmstadt, Germany) [10].

### Infection of mice

For infection, stationary-phase promastigotes were harvested and BALB/c mice were infected subcutaneously into one hind footpad with 3×10^6^ promastigote *L*. *major* parasites of the third to seventh *in vitro* passage.

### Preparation and culture of human and mouse macrophages and assessment of intracellular parasites

C57BL/6NCrl mice were purchased from Charles River (Sulzfeld, Germany) and maintained at the animal facility of University of Regensburg under pathogen free conditions. Female animals between 6 and 12 weeks of age were used for experiments. The preparation of thioglycolate-elicited peritoneal exudate macrophages was conducted, as previously described [11]. 200.000 thioglycolate-elicited peritoneal macrophages were cultured in 200μl RPMI 1640 medium (supplemented with 10% fetal calf serum (FCS), 100 units/ml Penicillin 100μg/ml Streptomycin (both PAN Biotech GmbH, Aidenbach, Germany), 50μM β-mercaptoethanol) in 96-well cell culture plates (BD Biosciences, Heidelberg, Germany). After 6 hours of incubation non adherent cells were removed.

hPBMCs were isolated from buffy coats by passage over a Leukocyte Separation Medium (PAA, Cölbe, Germany) gradient. The donors were healthy German adults without known exposure to *Leishmania* parasites. Human monocytes were isolated by exploiting their ability to adhere to plastic, subsequently human monocyte derived macrophages (hMDM) were generated as described elsewhere [10]. hMDM were used to proof the detection of living parasites by DAPI staining. For this human macrophages were infected with living (CFSE labeled) and co-incubated with dead and or apoptotic (SE-Alexa-Fluor-647 labeled) parasites for 3 hours with a ratio of 10 parasites per macrophage.

Mouse macrophage cultures were infected with different ratios of living *L*. *major* parasites (1:1 or 1:5). After 16 hours free parasites were removed by three washing steps with RPMI 1640 medium. To induce leishmanicidal molecules, macrophages were cultured for 72h in the presence or absence of IFN-γ (20 ng/ml, Fell, Germany). 50% of the cell culture media were replaced every 24h. Monocytes were stained with a solution of acridine orange (5 μg/ml) and ethidium bromide (50 μg/ml) for subsequent analysis by fluorescence microscopy as described before [12].

### PCR

To measure the parasite burden in infected macrophage cultures, genomic DNA was isolated using DNA purification solutions from QIAGEN (QIAGEN, Hilden, Germany). In brief, cells were digested in cell lysis solution directly in the well. Protein was removed by adding protein precipitation buffer and DNA was precipitated according to the manufacturer’s instructions with 100% 2-propanol (Sigma Aldrich, Taufkirchen, Germany). The concentration of mouse β-actin-DNA was quantified by PCR [13]. The *Leishmania* DNA concentrations in the same samples were determined using fluorescence resonance energy transfer real-time PCR with leishmanial 18S ribosomal DNA sequences [14]. The resulting *Leishmania* DNA copy number was then divided by the copy number of β-actin DNA to obtain the relative parasite density.

### Reagents, antibodies and immunofluorescent imaging of macrophages and parasites

The following antibodies were purchased from Fitzgerald Industries International (North Acton, USA): anti-CD11b (biotin-conjugated, clone M1/70) and anti-F4/80 (APC -conjugated, clone BM8). AF546-conjugated Streptavidin was bought from eBioscience (eBioscience, Frankfurt, Germany). 4,6-diamidino-2-phenylindole (DAPI) was obtained from Sigma Aldrich.

Cell cultures were blocked for 30 minutes with Cohn II at room temperature (50μl/well, 1 μg/μl; Sigma Aldrich). After washing, the cells were incubated for 1 hour with anti-CD11b and anti-F4/80 antibodies. Streptavidin was incubated for 45 minutes at room temperature. Nuclei from host cells and parasite-associated DNA were stained with DAPI (50μl/well, 1 μg/μl) after fixation with 4% PFA in PBS (100μl/well). Off note, only living parasites are stained with DAPI (S1B Fig). Ethidium bromide and acridine orange were delivered by Sigma. Thereafter, the stained cells were scanned with the TissueFAXSi-plus imaging system (TissueGnostics, Vienna, Austria) equipped with high-sensitivity 14 bit, 1,3 megapixel greyscale-digital PCO Pixelfly usb camera (PCO AG, Kehlheim, Germany). A series of separate images per fluorescence channel and field of view (FOV) were taken automatically, collected and merged afterwards to a virtual sample in TissueFAXSi-plus (acquisition software: TissueFAXS version 4.0.1.0128). Detailed instrumental settings will be given in the results section.

### Software-based analysis

We processed the images for the analysis of parasite numbers with TissueQuest or StrataQuest software (TissueGnostics). Using that software, three markers were applied: DAPI as master marker (nucleus) and AF546 (CD11b) as well as Cy5 (F4/80) as non-master markers. To achieve optimal cell detection the following parameters were adjusted: i) nuclei size; ii) discrimination by area; and iii) discrimination by grey. For the evaluation of positive cells scattergrams were created, allowing the visualization of corresponding positive cells in the source region of interest using the real-time back gating feature. The cut-off discriminates between false events and specific signals according to cell size and intensity of staining [15].

**Fig 1:**
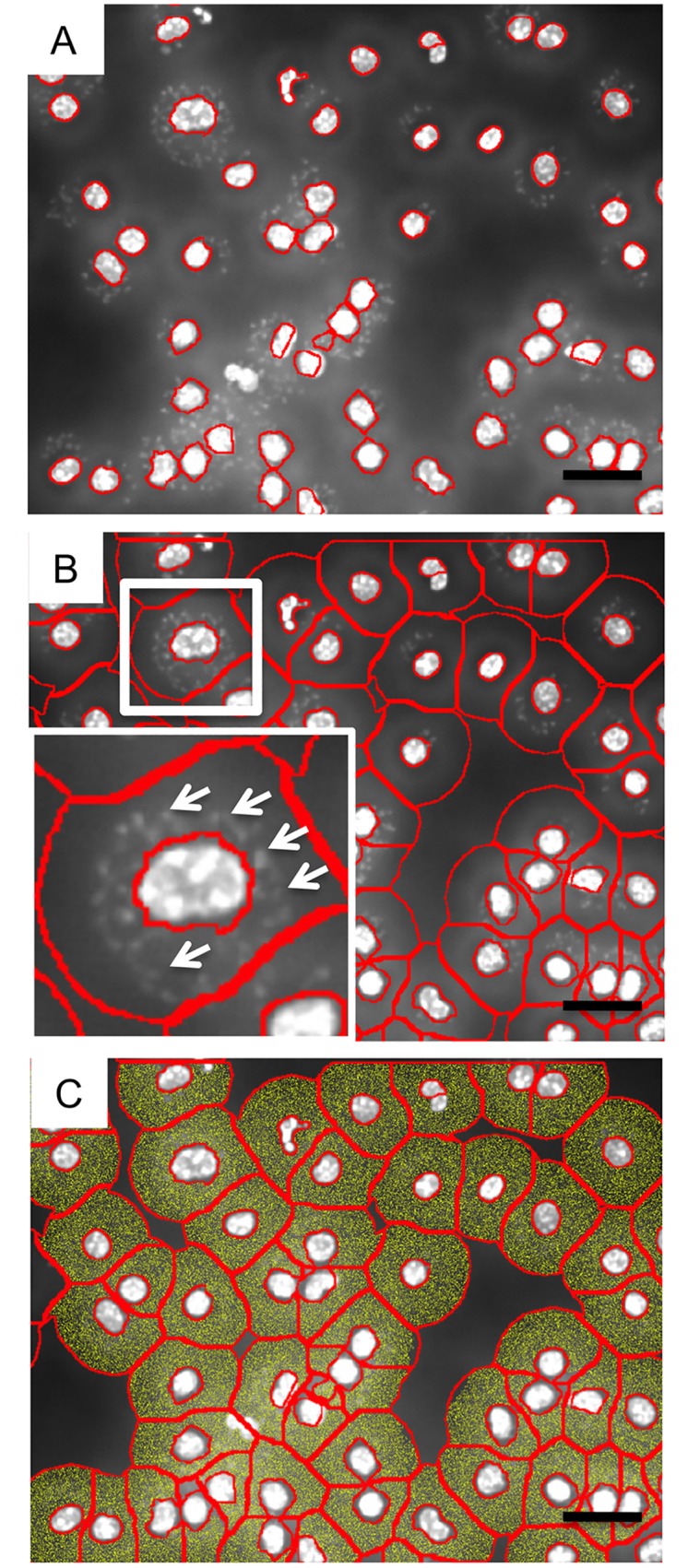
Nuclei detection and ring mask calculation by TissueQuest. Single channel greyscale pictures were processed by TissueQuest software. (A) The nuclei detection was performed according the following parameters (nuclei size: 10, remove small objects: 1, remove weakly stained objects: 1, automated background: no, automated threshold: 5, virtual channel: no, post processing order: remove, merge and Remove labels i) smaller then: 30 μm, larger then: 100 μm, weaker then: 137, stronger then: do not use; use merging rules: no). The detected nuclei are automatically surrounded by a red line. (B) The ring masks were created according the following parameters (interior radius: -0,31 μm, exterior radius; 12,74 μm, use identification cell mask: no, use nucleus mask: no, background threshold: 5; see also S1 Fig). The white insert highlights one infected macrophage. The white arrows depict some of the parasites within the ring mask. (C) The area within the ring mask is highlighted in yellow and represents the region of interest in which the screening of parasites can be performed automatically. Representative pictures out of 3 experiments are shown. Bar = 10μm.

For the detection of DAPI positive and living *L*. *major* parasites within the cells, a virtual channel for DAPI was created using a ring mask surrounding the cell nuclei. The *L*. *major* algorithm was applied only on the DAPI ring mask, excluding DAPI positive signals from the mammalian nuclei by slice exclusion (compare Fig 1). Detailed instrumental settings of the recommended Algorithm will be given in the results section.

### Preparation of lymph node sections for the detection of intracellular parasites

The skin-draining popliteal lymph nodes were embedded in optimal cutting temperature compound (OCT; Diatec, Hallstadt/Bamberg, Germany) and stored at –80°C. Tissue sections (6 μm) were thawed onto gelatin-coated glass slides, air-dried, fixed in acetone (for 5 min, at 4°C) and rehydrated with PBS and 0.01% Tween-20 (Sigma). Thereafter the sections were stained with DAPI as described above. After mounting with PermaFluor (Thermo Scientific, Dreieich, Germany), sections were analyzed by Axio Imager.M1 (Zeiss, Jena, Germany) equipped with high-sensitivity gray scale digital camera (AxioCam MRm, Zeiss). The gray scale images were processed with the StrataQuest software as described above.

### Statistical analysis

We used GraphPad Prism software for statistic analysis. Depending on the experimental setting the following tests were applied: i) nonparametric Mann-Whitney test (95% confidence interval) and ii) two-way ANOVA with Bonferroni post tests. The tests are indicated in the figure legends.

## Results

### Imaging and processing of pictures using the TissueFAXSi-plus workstation

96-well plates containing either *Leishmania*-infected macrophages or macrophages were included in our analysis. After fixation with 4% PFA a 4,6-diamidno-2-phenylindole (DAPI) staining was performed to visualize the overall chromatin structures, such as the mammalian nuclei and the protozoan DNA (kinetoplast and nucleus) [16]. This staining allows the detection of living parasites within macrophages because dead or apoptotic parasites are not able to uptake DAPI (S1 Fig).

Automated single-channel image acquisition was performed by the TissueFAXSi-plus system, which allows the fluorescence measurement using four channels (DAPI, FITC, Rhodamin, Cy5). Each single image contains approximately 50–200 cells that were stitched to one data set automatically by the TissueFAXS acquisition software (S2A Fig). Now the greyscale channels of interest can be processed in detail. According the parameters given by the TissueQuest software, nucleus detection must be performed. Settings of characteristics such as nuclei size, removal of small artificial objects and weakly stained objects is manually adjustable and recommended to use on every sample separately (S2B Fig). After that first setup, the software is able to detect DAPI-positive nuclei of mammalian cells (Fig 1A). To analyze potential parasites located within the cytoplasm of cells, the area around the nuclei is defined. Within this region the software screens for parasites (Fig 1B). The TissueQuest software solves that critical step by creating a ring mask. An algorithm creates a circle around the nucleus starting at the border of the detected DAPI stained nucleus. By defining the radius around the nucleus (approximately 12μm, see S2C Fig), the ring mask will not further increase beyond this value and also will automatically avoid overlap with adjacent ring mask (compare Fig 1B). The space between the nucleus and the ring mask border is highlighted in yellow (Fig 1C) and defines exactly the area that will be screened for the presence of *L*. *major* parasites.

### Detection of heterogeneous stained *L*. *major* parasites within the ring mask by TissueQuest

**Fig 2:**
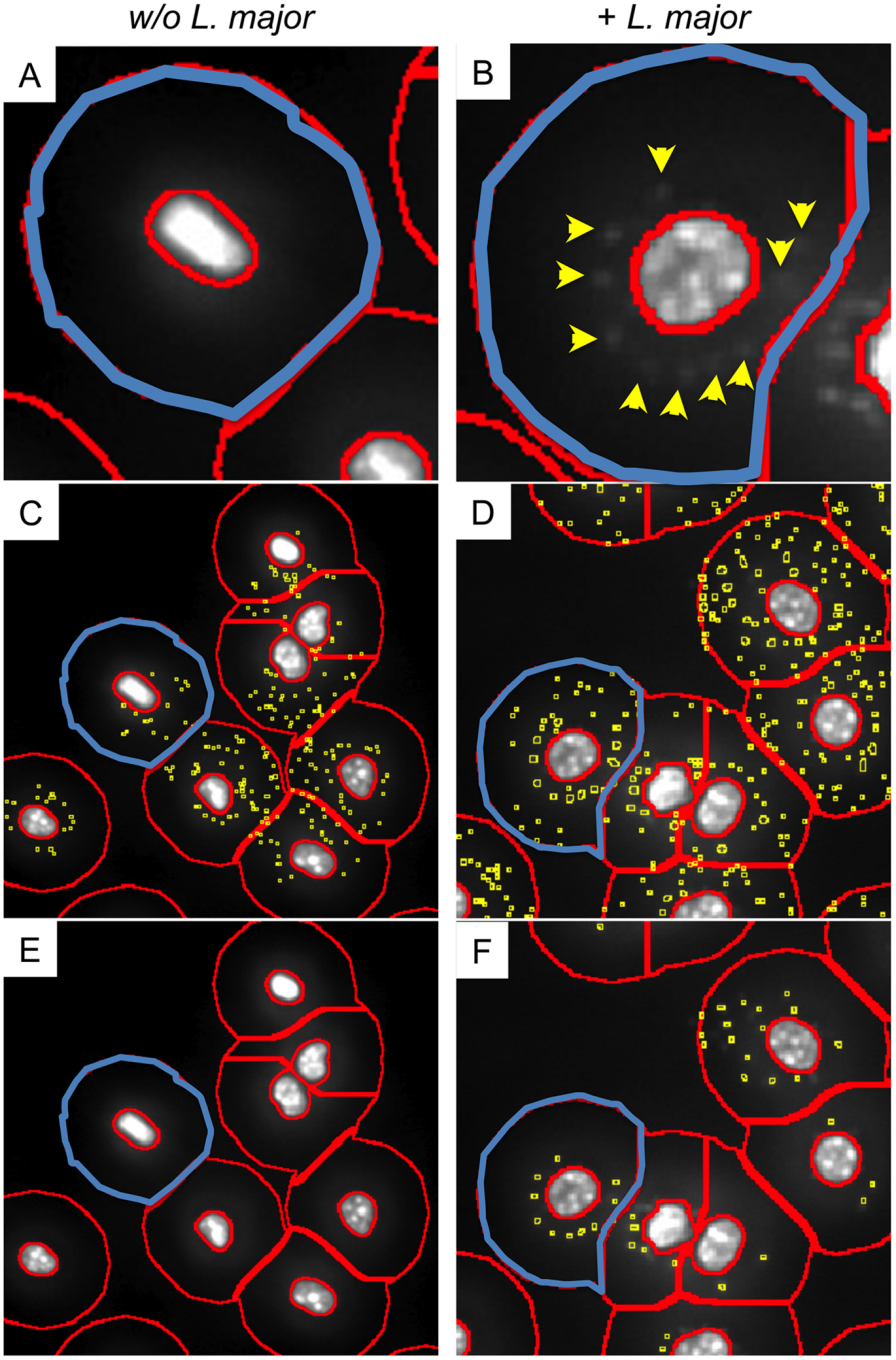
Detection of intracellular parasites within the ring mask. Macrophages were cultured in 96-well cultures in the presence (plus *L*. *major*, right row) or absence of *L*. *major* parasites (w/o *L*. *major*, left row). After 72h a DAPI staining was performed to visualize nuclei of the mammalian cells and the DNA rich areas of the parasites simultaneously. Single channel greyscale pictures were processed by TissueQuest software. The ring masks were created as described in Fig 1 and material and methods. (A) The ring mask (highlighted in blue) visualizes the cytoplasmatic area of a representative macrophage that was cultured in the absence of parasites. (B) One representative macrophage infected with *L*. *major* parasites is shown including the ring mask which is highlighted in blue. Visually determined DAPI positive signals are marked with yellow arrows. For the detection of parasites within the ring masks a virtual channel of the parasite-associated fluorochrome (in our case DAPI) has to be created (see S2C Fig). The following instrument settings were used: Use ring mask: yes, interior radius: -0,31 μm, exterior radius: 12,74 μm, use identification mask: no, use nucleus mask: no, background threshold: 5. (C) One algorithm developed for the detection of weak signals recognizes false positive signals (yellow squares) within the ring mask (the cell shown in A is highlighted in blue) of macrophages that are not infected. (D) False positive signals (yellow squares) are created. The representative (highlighted in blue) macrophage harbors more than 30 parasites, whereas no more than 10 can be visually determined (see B). (E) The algorithm that was developed to detect weak signals recognizes no false positive signals in the ring mask of macrophages that are not infected. (F) All parasites within infected macrophages are recognized (see yellow squares and yellow arrows in (B)). Representative pictures out of 3 experiments are shown. Bar = 10 μm.

According the very small DAPI signature of living parasites and the heterogeneous background within the ring masks (compare Fig 2A and 2B), new algorithms had to be developed suitable to recognize the parasites without giving false positive signals.

It is fairly not possible to create one single algorithm that fits for all staining protocols and cell types. Thus, the parasite finder software tool provides different algorithms which allow to choose an algorithm that exactly discriminates between the signal resulting from the pathogen and the background intensity within the ring mask.

Fig 2C exemplarily depicts an algorithm that was created for the detection of very weak parasite-derived signals. Based on the high sensitivity, a huge number of false positive signals within the ring masks of macrophages that were not infected with parasites appeared (Fig 2C). Consequently, background signals within the ring mask of infected macrophages are identified as false positive *L*. *major* signals (Fig 2D). Therefore, the user has to evaluate other algorithms on representative images to calibrate the system. An algorithm, which was created for the detection of strong *Leishmania*-derived signals gave correct results in the control group of not infected macrophages (Fig 2E). No parasites were detected within the ring mask of the macrophages that was formerly considered to be positive for *L*. *major* parasites (compare the highlighted cell in Fig 2C and 2E). Furthermore, the algorithm detects *L*. *major*-positive signals within the ring mask of infected macrophages (see blue-highlighted cell Fig 2F). The software driven detection of the parasites is congruent with the visually identified *L*. *major* parasites (compare Fig 2B, yellow arrows and Fig 2A blue ring mask).

From this data we conclude that the algorithm is suitable for the detection of intracellular living pathogens. However, depending on the quality of images, a precise instrument setting including all necessary controls is crucial to avoid the generation of false negative and false positive data.

### Quantification of parasite density within infected macrophages and infection rate by TissueQuest

**Fig 3:**
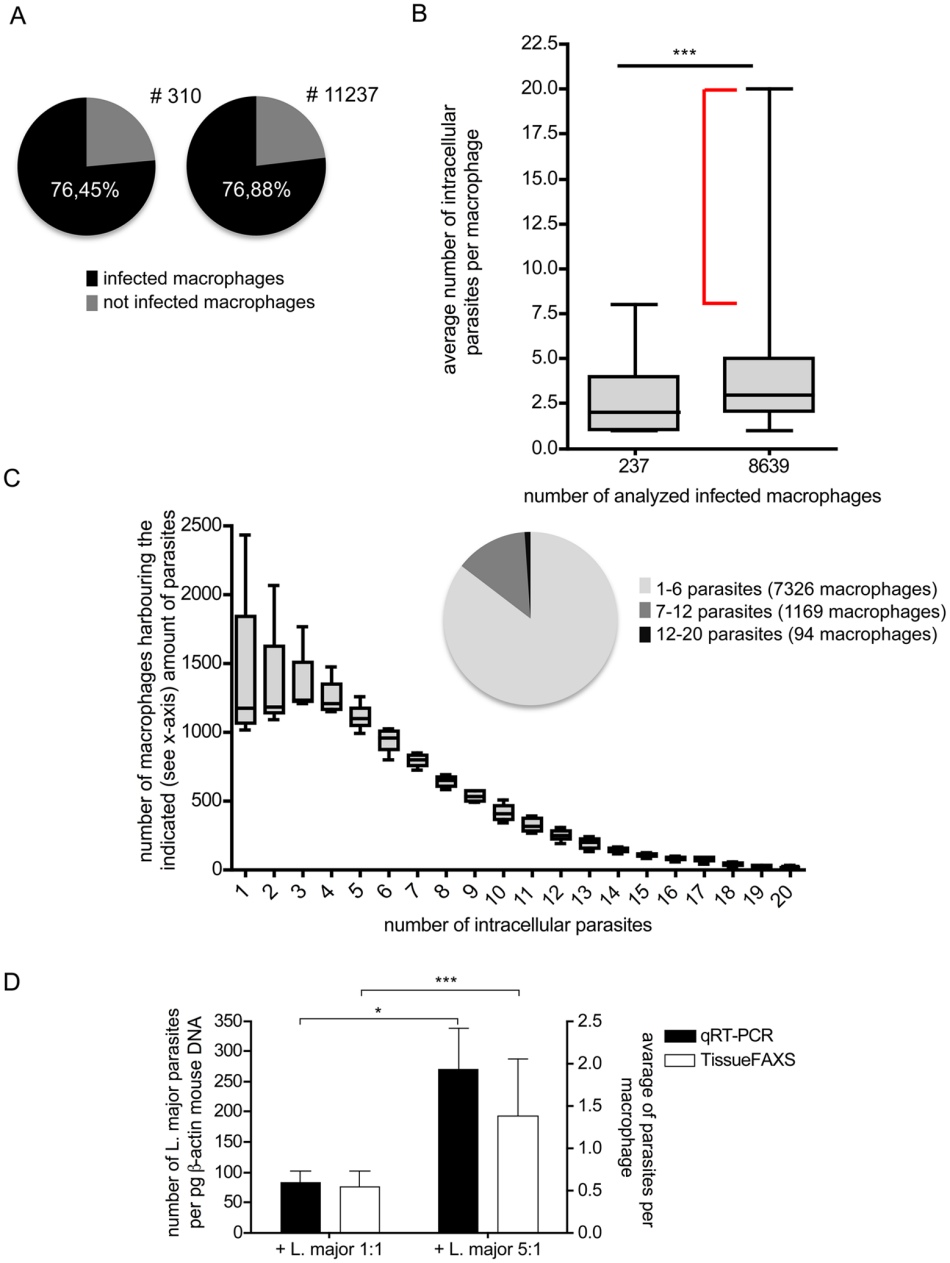
TissueQuest-based characterization of infected macrophages. Macrophages were cultured in 96-well plates in the presence of *L*. *major* parasites (parasite/macrophage ratio 5:1). (A) The infected macrophages were determined automatically by the TissueQuest software. The circle diagrams depict the distribution of infected (black) and not infected macrophages (grey). The infection rate is indicated within the circle diagram. A representative experiment visualizing the infection rate after measuring 310 or 11237 macrophages is shown. (B) The box-plots depict the TissueQuest based quantification of the average number of intracellular parasites per macrophage after analysis of 237 or 8639 macrophages. The gray boxes indicate the range between the first and third quartile. The horizontal lines indicate the median. The whiskers visualize the spread of data. The red bracket highlights the additional information that was generated after measuring 8639 infected macrophages compared to analyzing 237 infected macrophages (***<0.0001). The data shown in (A) and (B) are representative for three experiments. (C) Macrophages were analyzed regarding their number of intracellular parasites and the amount of macrophages (y-axis) harboring the indicated number of parasites (x-axis) are shown. Data are presented in box-blots. The circle diagram highlights the number of macrophages harboring 1–6 (light gray), 7–12 (dark gray) and 12–20 (black) parasites. Data are representative for three experiments. (D) Validation of TissueQuest-based quantification of parasites by real-time PCR was performed. Macrophages were infected with different numbers of parasites per host cell (1:1 and 5:1) and 96 hours post infection the average number of parasites per macrophage (right y-axis) and the number of *L*. *major* parasites per β-actin was determined (left y-axis) (*<0,01, *** 0,0001; n = 3; mean +/- SD).

The TissueQuest software enables a simultaneous detection of DAPI positive macrophage nuclei and DNA from living amastigote parasites within the non communal parasitovorous vacuols [17]. To further evaluate the sensitivity of TissueQuest, we analyzed the infection rate of macrophages that were co-cultured with *L*. *major* parasites *in vitro*. Analyzing 310 cells within one selected region of interest the software calculated an infection rate of 76,45% (Fig 3A). Furthermore, we were interested to verify whether the calculated infection rate becomes more precise after the analysis of more than 10.000 cells. As shown in Fig 3B the infection rate of 11237 cells (76,88%) is comparable with the infection rate calculated after the measurement of 310 cells. Thus, we can conclude that a random sample is sufficient to generate valid data concerning the overall infection rate.

Next we calculated the average number of parasites within each macrophage and validated the accuracy of a randomly chosen sample. As shown in Fig 3B the median number of parasites within the macrophage culture is 2.0 after analyzing 237 macrophages. In contrast, the median number of parasites within the macrophage is 3.0 when 8639 macrophages were analyzed (Fig 3B). Thus, the examination of 8639 infected macrophages gives a more precise result regarding the variance of the number of parasites within the macrophages. Furthermore, the quantification of the parasitic load of each single macrophage elucidated the differences obtained from the analysis of 237 (infection rate: 1 to 8 parasites) and 8369 macrophages (infection rate: 1 to 20 parasites; Fig 3B).

Consequently, the information about the population of macrophages infected with more than 8 parasites (see red bracket in Fig 3B) was lost in case of measuring only 237 macrophages.

Based on the data generated by the TissueQuest software, it is clearly evident that most of the macrophages (7326 out of 8639) are infected with 1 to 6 parasites (compare Fig 3C circle diagram). Macrophages with 7–12 parasites (1169 out of 8639) as well as macrophages with 12–20 parasites (94 out of 8639) represent only a minor population (Fig 3C). Thus, TissueQuest can be used to analyze i) the overall infection rate, ii) the distribution of intracellular parasites within macrophages, and iii) the categorization of the number of macrophages with a distinct parasite load.

To further investigate the comparability of the TissueQuest-generated data with well established methods, the parasite burden of infected macrophages was also evaluated with real time PCR analysis. For this purpose macrophages were infected with a ratio of 1 parasite per macrophage (1:1) or 5 parasites per macrophage (5:1). This was done to create macrophage cultures with different parasite loads. Multiple washing steps removed free parasites, to avoid the falsification of the data by contamination of extracellular DNA. As expected, real time PCR analysis revealed a higher parasite load in cultures that were infected with the ratio 5:1 compared to the lower parasite macrophage ratio of 1:1 (Fig 3D). Similar results were archived by the TissueQuest software (Fig 3D) and thereby the sensitivity of the new technology could be verified.

### Quantification of myeloid cell markers expressed by infected and non-infected macrophages *in situ*

Based on the TissueQuest analysis we could demonstrate that the distribution of living parasites within macrophages is highly heterogeneous (Fig 3). The reason for that different parasite numbers per macrophages can be attributed to diverse phagocytosis activities and host cell-associated factors potentiating the proliferation of parasites. Thus, it might be possible that the macrophage phenotype determines the expansion of intracellular parasites. Consequently, it would be very informative to generate data correlating the parasite numbers and expression intensities of distinct markers used for the identification of cell subsets.

Depending on the microscope configuration and the equipped filters, different markers can be analyzed in combination. In the following experiments, adherent cells were stained with DAPI for host cell nuclei and parasites DNA detection and markers commonly used for macrophage classification (the integrin α_M_ chain CD11b and F4/80 [18]) were applied.

**Fig 4:**
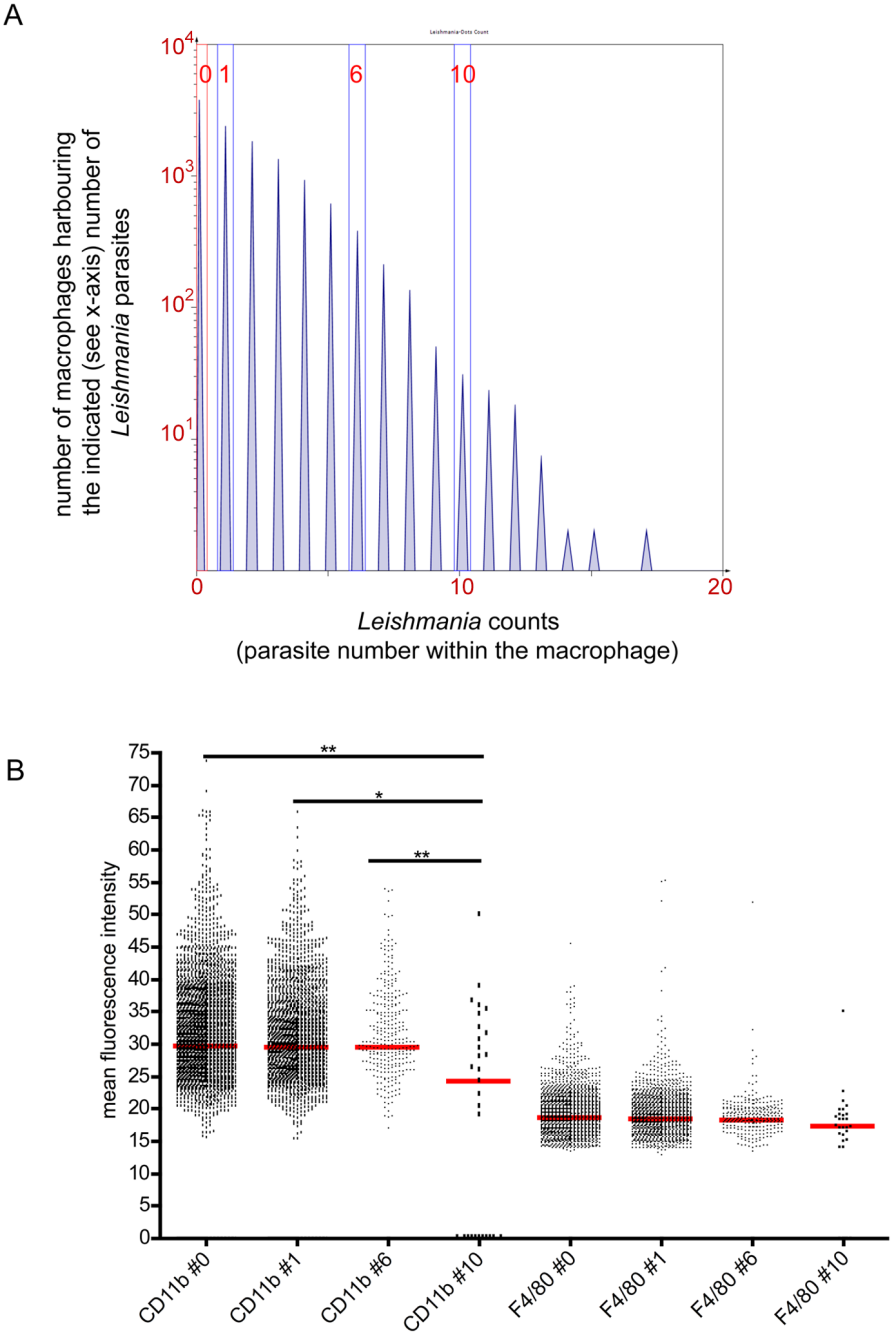
Phenotypical analysis of macrophages harboring different numbers of parasites. Macrophages were cultured in 96-well plates in the presence of *L*. *major* parasites (parasite/macrophage ratio 5:1). After 72 hours the cells were stained with F4/80^Cy5^, CD11b^biotinylated^ + Streptavidin^AF546^. Macrophage nuclei and parasite DNA rich areas were stained with DAPI. (A) The TissueQuest-based analysis of cells and intracellular parasites are presented. A histogram plot depicts the number of parasites within the macrophages (x-axis) and the number of macrophages harboring the indicated (see x-axis) number of *Leishmania* parasites. Cells within the highlighted gates were further analyzed regarding the expression of CD11b and F4/80. (B) The mean intensities of CD11b or F4/80 are plotted representing macrophages harboring no (#0), one (#1), six (#6) or ten (#10) parasites. Statistical analyses were performed with GraphPad Prism using the nonparametic Mann-Whitney test (p**<0,01, p*<0,05; red horizontal line represents the median). Every single dot represents one individual analyzed nucleated cell. One representative set of data out of two experiments is shown.

96-well plates were processed automatically and single grey scale pictures including the information about i) parasite load, ii) expression of CD11b and iii) expression of F4/80 were generated. Based on that set of data the TissueQuest software can evaluate every single nucleated host cell *in situ* regarding the amount of intracellular parasite (Fig 4A).

Now it is possible to compare the expression of distinct markers by macrophages harboring different numbers of parasites. After the incubation of macrophages with parasites the infection rate is highly heterogeneous (compare Fig 3). As shown in Fig 4A the TissueQuest software can generate a histogram plot showing the number of parasites within the macrophages (x-axis) and the number of macrophages harboring a distinct number of parasites (y-axis). We decided to compare macrophages harboring 0, 1, 6, and 10 parasites (gates were created accordingly (Fig 4A)). The TissueQuest software detects the following parameters: i.) nuclei, ii.) ring mask, iii.) parasites within the ring mask, and iv.) expression intensity of CD11b and/or F4/80 within the ring mask. At a first glance the mean intensities of CD11b and F4/80 demonstrate a high variation (Fig 4B). Statistic analysis revealed that there is no normal distribution of the CD11b and F4/80 intensities within the indicated population which indicates that the expression of CD11b and F4/80 by infected and non-infected macrophages is very heterogeneous. Furthermore, our data demonstrate that CD11b expression correlates with the parasite number: the higher the number of parasites within the macrophages, the lower is the mean intensity of CD11b. The molecular mechanisms are so far unknown and will be analyzed in future studies. In contrast the mean expression of F4/80 does not show significant differences between macrophages that are infected with one, six, ten or no parasites.

### Evaluation of living and dead intracellular parasites *in vitro* and *in situ*

DAPI staining is useful for the detection of living parasites within macrophages (S1 Fig), which allows the investigation of leishmanicidal activities of macrophages. In line with already published studies [19] our algorithm confirms that stimulation of macrophages with IFN-γ results in a reduction of living parasites *in vitro* (S3 Fig).

**Fig 5:**
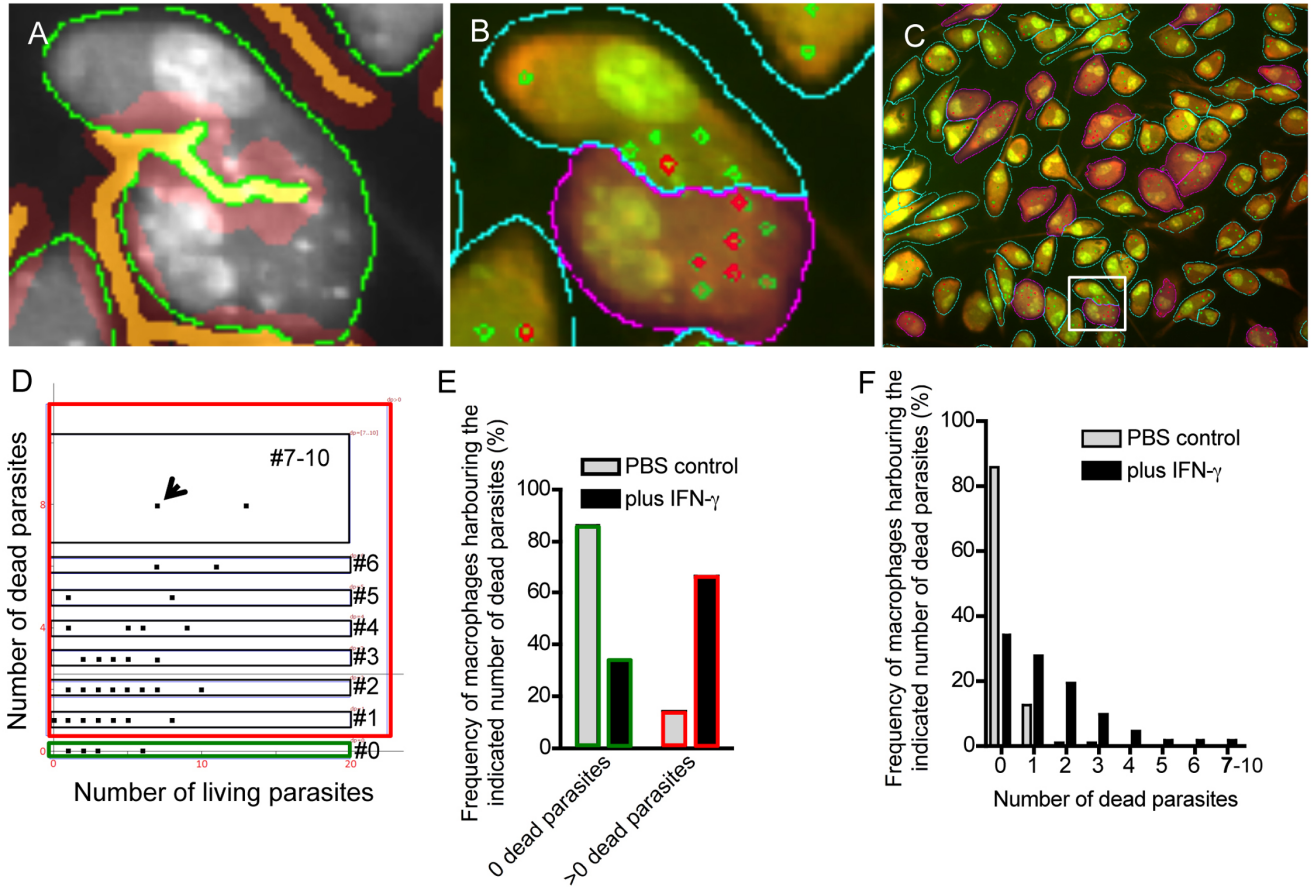
Quantification of living and dead cells *in vitro*. Macrophages were pulse infected with *L*. *major* parasites and cultured in the presence or absence of IFN-γ (20 ng/ml) for 72h. Cells were stained with EB-AO and analyzed by fluoresence microscopy. Single gray scale pictures were acquired in the EB and AO channel. (A) The AO gray scale image was used for membrane detection algorithm (green line) and the cell border identification (orange line). (B) Based on the cytoplasm (blue line) and cell border detection, it is possible to dissect cells that stick together adequately. The separation is highlighted in magenta. The parasite algorithm can detect dead (EB channel) and living (AO channel) parasites within the cytoplasm in parallel. Dead parasites are highlighted in red, living parasites in green. (C) A backward connection gating strategy was used to identify macrophages harboring more than 2 dead parasites (highlighted in magenta). D) Macrophages stimulated with IFN-γ are shown. The dot plot diagram visualizes a pool of macrophages (shown as a single dot) with a distinct number of dead (y-axis) and living (x-axis) parasites. The green gate highlights macrophages harboring only living parasites. The red gate highlights macrophages with one or more dead parasites. The black gates refer to macrophages infected with 1, 2, 3, 4, 5, 6, or 7–10 parasites. (E) Analysis of the frequency of macrophages harboring 0 (green gate) or more than 0 (>0, red gate) dead parasites. The macrophages were stimulated with IFN-γ (black bars). PBS controls (gray bars) represents macrophages that were not stimulated. (F) Analysis of the frequency of macrophages harboring a distinct number of dead parasites (x-axis). The macrophages were stimulated with IFN-γ (black bars) or served as controls (PBS; gray bars).

Dead parasites are not able to uptake DAPI and therefore TissueQuest is able to differentiate between living and dead parasites inside of the host cells. To confirm these results ethidium bromide acridine orange (EB-AO) staining was performed in parallel [19]. The StrataQuest program detects the cytoplasm of cells (Fig 5A, green line) and the cell-cell contact region (Fig 5A, orange). Consequently, cells that are attached to each other can be dissected precisely (Fig 5B). Furthermore, StrataQuest can evaluate the EB (red) and AO (green) intensities of parasites within the cytoplasm (Fig 5B). According to the backward connection function, it is possible to highlight macrophages with a distinct number of living or dead cells. This is important to consider the question of whether all macrophages show the same leishmanicidal activities.

Fig 5B exemplarily depicts a cell (highlighted in magenta) that harbors more than two dead parasites. All cells within the region of interest showing the same characteristic (more than two dead parasites), can be highlighted by the program (Fig 5C). Macrophages that are not stimulated with cytokines inducing leishmanicidal molecules are hardly able to kill parasites (S3 Fig). In contrast IFN-γ stimulation induces the pool of macrophages with more than 2 dead parasites (Fig 5C, highlighted in magenta). A detailed statistic analysis comparing dead versus living parasites can be performed. We decided to compare the number of dead (y-axis) and living parasites (x-axis) within the macrophage population (Fig 5D). The dots in the diagrams depict a pool of macrophages with different characteristic. The arrow in Fig 5D highlights a pool of macrophages with 6 living and 8 dead parasites. According the chosen gating procedure, statistically analysis can be performed. Fig 5E depicts the frequency of macrophages harboring 0 dead parasites (all parasites within the cells are alive). The frequency of this pool of host cells decrease from 85,89% to 33,91% after stimulation with IFN-γ representing the induced killing activity.

In addition, the software can characterize the frequency of macrophages harboring a distinct number of dead parasites (Fig 5F). As expected the frequency of host cells harboring dead parasites increases after IFN-γ stimulation.

**Fig 6:**
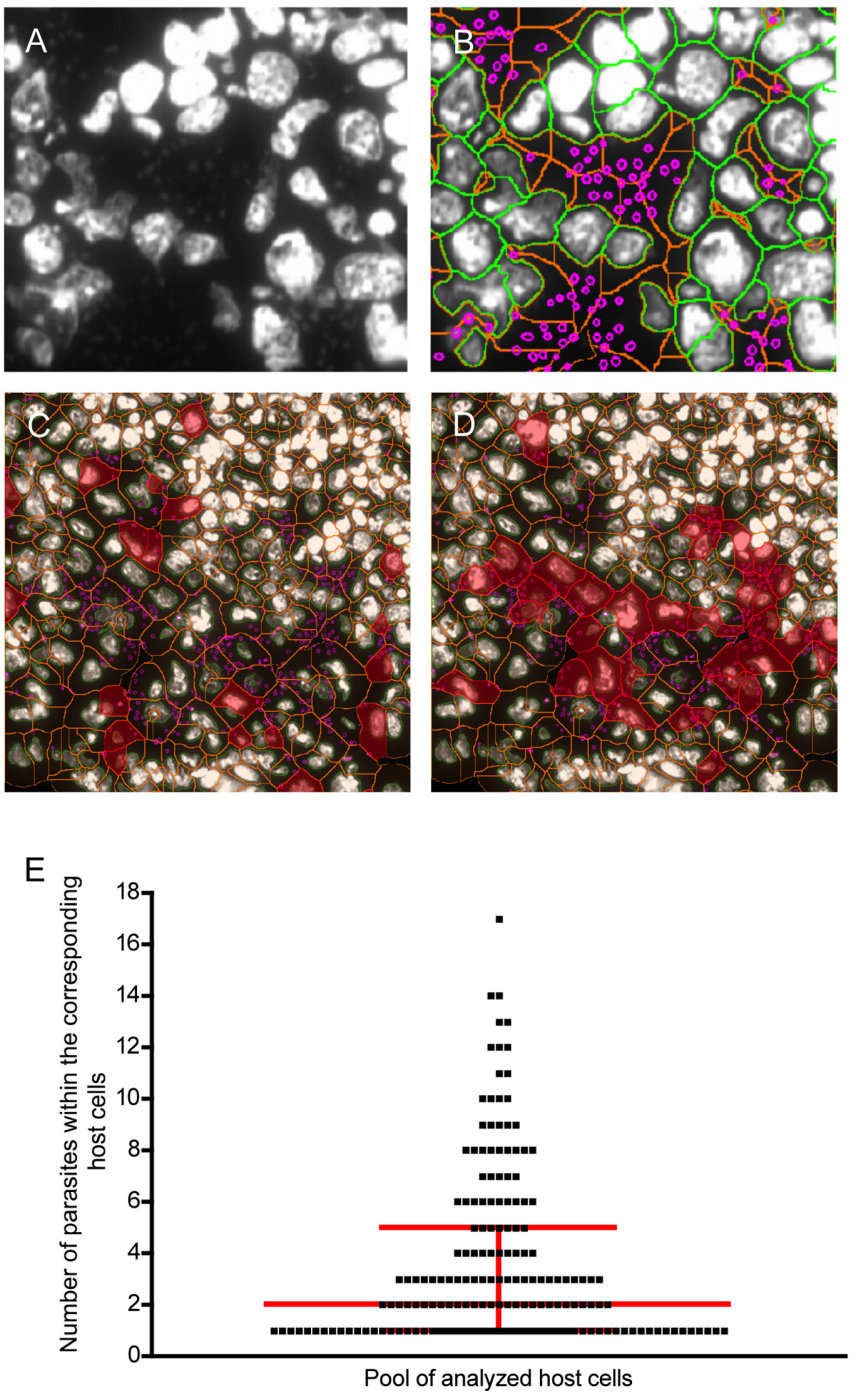
Assessment of parasite load *in situ*. The skin-draining lymph nodes of infected BALB/c mice were removed 20 days after infection. Cryosections were stained with DAPI and analyzed by fluoresence microscopy. (A) One representative region of the infected lymph node is shown. The gray scale image depicts DAPI^+^ host cell nuclei and parasite DNA. (B) An overlay of the detection of nuclei (green), cytoplasm (orange), and parasites (magenta) is shown. (C) Backward gating visualizes cells harboring 3 and (D) 5–10 parasites (highlighted in magenta). E) The graph represents the parasite load (y-axis) within infected cells. The horizontal line represents the median and the arrow bars display the interquartile range. Each dot represents a host cell harboring the indicated (y-axis) amount of parasites.

Next, we investigated the possibility to differentiate live and dead parasites *in situ* and analyzed infected skin-draining lymph nodes of BALB/c mice. Cryosections were stained with DAPI and visualized by the TissueQuest software (Fig 6A). DAPI signals were measurable indicating that the parasite DNA was not degraded. This in turn suggests that the visualized parasites were alive before biopsy processing, because dying or dead parasites cannot be stained with DAPI (S1 Fig). The StrataQuest software creates a cytoplasmatic (Fig 6B, orange, line) and a nuclei mask (Fig 6B, green line). Therefore, the DAPI^+^ parasites can be detected within the cytoplasmatic area (Fig 6C, magenta, circles) of the host cell. After data acquisition statistical analysis and backward gating was performed.

We decided to visualize exemplarily the cells harboring 3 parasites (Fig 6C) and 5–10 (Fig 6D). It is obvious that the cells with 3 parasites are scattered within the section whereas the cells harboring 5–10 parasites aggregates in the T-cell area in the lymph node. A statistical analysis including parameters such as i) infection rate, ii) median number of parasites within host cells, iii) identification of the interquartile range of infection can be performed. Our representative analysis of 387 cells revealed that 53,49% of the nucleated cells are infected. The interquartile range is 1–5 parasites per cell. The median is 2,0 (Fig 6E). All these information are useful to characterize the spreading of parasites and the host parasite interaction.

In conclusion, we demonstrated that the software can detect and quantify parasites in infected tissues. Thus, it is possible to identify the localization of host cells harboring a distinct number of parasites. Additionally, the phenotyping (as described in Fig 2) will give information regarding the subset of infected myeloid cell.

## Discussion

Over the last decades it turned out that multi-parameter analysis are crucial for the exact determination of myeloid cell subsets [1]. In this regard immunofluorescent-based cytometry analysis has developed to an emerging field. One reason for this tendency is certainly that the immunofluorescent-labeled antibodies can detect several parameters simultaneously. In addition, the development of highly sophisticated analysis software was propelled. However, classical flow cytometry-based analysis of single cell suspensions shows technical limitations in case of quantification of the intracellular parasites and phenotyping of the corresponding host cell. Innovative concepts such as ImageStream that can take images of cells within the sheath fluid are unsuitable to give details regarding a possible cell-cell interaction and the localization *in situ*. Consequently, further improvements in the field of visualization and phenotyping of cells are needed.

In our study we present a new method to characterize infected host cells *in vitro* and *in situ*. In contrast to existing open source software INsPECT that combines standard image analysis commands to generate a certain level of automation and data accuracy, TissueQuest and StrataQuest software represent a dedicated and quality controlled approach to single cell identification and subsequent intra- as well as extracellular analyses [20]. By applying standard image processing commands (e.g filter-, threshold- and binary morphology operations, edge detectors, Watershed algorithms) used in most open source or freeware image analysis software (eg. ImageJ, INsPECT) dense cell populations cannot be properly segmented. Thus, more elaborated high-level methods have to be applied [21, 22]

The functionality of the presented software depends on the quality of the fluorescence signature of mammalian nuclei and parasite DNA. This is of particular importance, because parasite-derived DAPI signal is very low compared to the bright DAPI signal of mammalian nuclei. Additionally the intensities of nuclei- and parasite-derived DAPI signals can be heterogeneous within one probe. Thus the software has to differentiate between the real parasite-derived signal and random generated background noise. To achieve this accuracy, TissueQuest and StrataQuest provide different “Dot Finder” algorithms. These programs vary in terms of sensitivity and detection limit and allow for the first time the discrimination between host cells and parasites in a suitable manner. Moreover, our data revealed that apoptotic or dying parasites are incapable to incorporate DAPI and therefore specific algorithms are able to differentiate between live (DAPI positive) and dead parasites (DAPI negative). Consequently, the TissueFAXS-based approach can be used for high throughput analysis of factors driving leishmanicidal activities of infected macrophages.

In addition, EB-AO in combination with StrataQuest analysis can be applied in situ to discriminate between living and dead pathogens within the host cells and allow the application of different statistical analysis. Given the fact that *L*. *major* amastigotes replicate within the non communal parasitovorous vacuols (PVs) [17], it is now possible to identify and characterize those vacuoles which contain leishmanicidal molecules. Off note, GFP or YFP expressing microbes can be traced *in vitro* and *in situ* as well and thereby allow the investigation of e.g. *Mycobacteria* and *Listeria*, which display a week DAPI signature [23–25].

Besides the detection and quantification of internalized parasites in macrophages, it is also now feasible to analyze the influence of the parasite on the phenotype of the infected cells in general but also dependant of the parasitic burden in each single cell. Our first performed experiments elucidated that some cell type specific markers are influenced by the amount of intracellular parasites. For example, CD11b was down regulated in correlation to an increased parasitic burden. These findings will be investigated in more detail and might help to understand the molecular mechanisms leading to parasite control or replication.

Another advantage is the automation and as a result the high throughput option of TissueFAXS analysis. Several hundred pictures in a short period of time can be acquired and analyzed. In this context we could show that analyzing hundreds of cells is sufficient to create a precise statement regarding the infection rate. However, for exact evaluation of the number of parasites within the host cells ten “thousands” of infected macrophages have to be screened by the software which is not possible to achieve by manual counting.

As mentioned above, the algorithm is able to detect parasites not only *in vitro* but also *in situ* and thereby allows the investigation of cell-cell interaction and cell localization inside of an organ. Tissue samples from patients or animal models can now be characterized on a new level. Using OCT-compound embedding medium, host and parasite DNA and proteins a cry conserved and represent nearly the “vital” situation. This in turn allows staining the intact DNA of “living” parasites and hosting cells with DAPI and the subsequent analysis with StrataQuest and TissueQuest. Due to the high density of the tissue it is necessary to define the cytoplasm of neighboring cells. Special tools, such as membrane and nucleus detection in the software allow this definition. With the backward gating function we were able to find out that cells harboring 7–10 parasites aggregate in distinct areas of the tissue. The biological function is not known at the moment but might be of importance to improve therapy strategies in the future.

In conclusion, TissueQuest and StrataQuest software for high throughput microscope-based multicolor cell analysis enable the characterization of i.) parasitic burden as well as the ii.) phenotype of the infected cells in addition to iii.) its localization and iv.) cell-cell interaction. Future studies using this technology will allow the detailed investigation of molecular mechanism in host-parasite interaction and thereby might help to improve therapeutic strategies combating microbial diseases.

## Supporting Information

S1 Fig Identification of living parasites with DAPI. Dying parasites (A) or living stationary phase parasites (B) were harvested from blood agar plates. After labeling with CFSE an additional DAPI staining was performed (CFSE (green) and DAPI (blue)). (C) Living stationary phase parasites *L*. *major* parasites were labeled with CFSE. Dying parasites were labeled with SE-Alexa-Fluor-647. Human macrophages were infected with a 1:1 ratio of living and dead parasite samples. DAPI staining was performed 90 min after infection to highlight intact DNA.
(EPS)

S2 Fig Representative screenshots from the TissueQuest analysis software. Macrophages cultured within 96-well plates were stained with DAPI. Thereafter single pictures were automatically generated using the TissueFAXSi-plus analysis system. (A) The overview visualizes the menu bars and the tools required for the TissueQuest-based analysis of acquired pictures. (A.I) The project browser gives detailed information regarding the samples that were acquired. (A.II) Details such as name, sample, rows count (vertical number of the stitched images), columns count (horizontal number of the stitched images), FOVs count (number of field of views), excluded FOVs count (excluded number of field of views), area of analysis are documented. (A.III) The stitched image is shown. The white squares highlight the stitched individual single pictures (n = 83). The orange circle (8.779 mm^2^) indicates the region of interest that was chosen for analysis. A.IV) This insert depicts the analyzed region of interest (orange circle in A.III). (B) The settings for the DAPI channel (nuclei of macrophages) are shown. (C) Parameters for Ring mask detection. Within the defined area (Ring mask) the Dot-finder detects the DAPI positive DNA rich areas (nucleus and kinetoplast) of the parasites.
(EPS)

S3 Fig Determination of leishmanicidal activities by TissueQuest based analysis. Macrophages were cultured for 72h in 96-well plates in the presence of *L*. *major* parasites (parasite/macrophage ratio 5:1). The cells were stimulated with IFN-γ (20ng/ml) or PBS (negative control). The number of DAPI^+^ parasites was determined by TissueQuest analysis using the dot finder algorithm. Each dot represents the average number of living parasites per cell (PBS, n^macrophages^ = 17196, IFN-γ, n^macrophages^ = 90586). The horizontal line indicates the median. Pooled data from three experiments are shown (p***<0,0003).
(EPS)
